# Rituximab Therapy in Renal Amyloidosis Secondary to Rheumatoid Arthritis

**DOI:** 10.3390/biom8040136

**Published:** 2018-11-05

**Authors:** Levent Kilic, Abdulsamet Erden, Yusuf Ziya Sener, Berkan Armagan, Alper Sari, Umut Kalyoncu, Omer Karadag, Ali Akdogan, Ismail Dogan, Sule Apras Bilgen, Sedat Kiraz, Ihsan Ertenli

**Affiliations:** 1 Department of Internal Medicine, Division of Rheumatology, Hacettepe University Faculty of Medicine, 06100 Ankara, Turkey; drleventkilic@yahoo.com (L.K.); berkanarmagan@gmail.com (B.A.); snalpersari@hotmail.com (A.S.); umut.kalyoncu@yahoo.com (U.K.); omerkaradag@ymail.com (O.K.); aakdogan@hacettepe.edu.tr (A.A.); dridogan@hotmail.com (I.D.); sapras@hacettepe.edu.tr (S.A.B.); skiraz@hacettepe.edu.tr (S.K.); iertenli@hacettepe.edu.tr (I.E.); 2 Department of Internal Medicine, Hacettepe University Faculty of Medicine, 06100 Ankara, Turkey; yzsener@yahoo.com.tr

**Keywords:** rituximab, amyloidosis, rheumatoid arthritis, biologic registry, proteinuria

## Abstract

Secondary amyloid A (AA) amyloidosis is a late and serious complication of poorly controlled, chronic inflammatory diseases. Rheumatoid arthritis (RA) patients with poorly controlled, longstanding disease and those with extra-articular manifestations are under risk for the development of AA amyloidosis. Although new drugs have proven to be significantly effective in the treatment of secondary AA amyloidosis, no treatment modality has proven to be ideal. To date, only in small case series preliminary clinical improvement have been shown with rituximab therapy for AA amyloidosis secondary to RA that is refractory to TNF-α inhibitors (TNF-i) therapy. In these case series, we assessed the efficacy and safety of rituximab therapy for patients with RA and secondary amyloidosis. Hacettepe University Biologic Registry was developed at 2005. The data of the RA patients who were prescribed a biological drug were recorded regularly. Patients with biopsy proven AA amyloidosis patients were screened. Of 1022 RA patients under biologic therapy, 0.7% patients had clinically apparent histologically confirmed amyloidosis. Four of seven patients who were prescribed rituximab at least one infusion enrolled to those case series. Two of four patients showed significant clinical improvement and one of them also had decrease in proteinuria and the other one had stable renal function and proteinuria. The main goal for the treatment of AA amyloidosis is to control the activity of the underlying disorder. In this study, we showed that rituximab may be an effective treatment in RA patients with amyloidosis who were unresponsive to conventional disease modifying anti-rheumatic drugs (DMARDs) and/or TNFi.

## 1. Introduction

Amyloidosis is a disorder of protein folding in which toxic insoluble β-sheet fibrillar protein aggregates that progressively disrupt tissue structure and function. Amyloidosis can be acquired or hereditary. The disease can be localized or systemic. The most common causes of amyloidosis are the immunoglobulin-light-chain relate amyloidosis (AL), amyloid transthyretin (ATTR) amyloidosis, and reactive (secondary) amyloidosis (AA) due to chronic inflammatory diseases like chronic infections and rheumatoid arthritis (RA). Secondary AA amyloidosis is a complication of chronic inflammatory disorders that gives rise to overproduction of the acute-phase reactant serum amyloid A protein (SAA). The AA amyloid fibrils are composed of AA protein, an N-terminal fragment of SAA which is a prerequisite for AA amyloid formation. Most SAA in plasma is produced by hepatocytes under transcriptional regulation by cytokines, especially interleukin-1 (IL-1), IL-6, and tumor necrosis factor (TNF). Its circulating concentration can rise from normal levels with an acute inflammatory stimulus and can remain persistently high in chronic inflammation [1,2,3].

Secondary amyloidosis is a late and serious complication of poorly controlled, chronic inflammatory diseases [1]. Seropositive RA patients with poorly controlled, longstanding disease and those with extra-articular manifestations are under risk for the development of AA amyloidosis [4,5]. Post-mortem incidence of amyloid in RA patients has ranged from 10 to 25 percent, similar values of 11 to 29 percent have been found in living patients with RA, depending on population and diagnostic strategy [6]. However, the prevalence of clinically symptomatic amyloidosis was much lower that has ranged from 2 to 11 percent, with considerable variation between geographic areas [7,8,9]. Systemic AA amyloidosis can cause significant mortality due to end-stage renal disease and infections. Although new drugs have proven to be significantly effective in the treatment of secondary AA amyloidosis, no treatment modality has proven to be ideal. The suggested treatment of AA amyloidosis secondary to chronic inflammatory diseases is to suppress inflammation of underlying disease [4]. Recently, several isolated cases and small series have demonstrated therapeutic approaches focusing on TNF-α inhibitors (TNF-i) therapy or tocilizumab have achieved significant clinical improvement and partial resolution of AA amyloid deposits in RA patients [10,11,12]. Rituximab, an anti-CD20 monoclonal antibody, is efficacious for patients with severe active RA who have exhibited an inadequate response classical disease modifying anti-rheumatic drugs (DMARDs) and TNF-i. However, to date, only in small case series preliminary clinical improvement has been shown with rituximab therapy for AA amyloidosis secondary to rheumatoid arthritis that is refractory to TNF-i therapy [13,14]. 

In these case series, we assessed the efficacy and safety of rituximab therapy for patients with RA and secondary amyloidosis. 

## 2. Materials and Methods

### 2.1. Patients Selection

Hacettepe University Biologic Registry (HUR-BIO) was developed at 2005. The data of the RA patients who were prescribed a biological drug were recorded regularly. Patients with biopsy proven AA amyloidosis patients were screened. Seven (0.7%) RA patients had AA type amyloidosis. Four of seven patients who were prescribed rituximab at least one infusion enrolled to those case series. 

### 2.2. Collected Data of Biological Dataset

The HUR-BIO registry included demographic characteristics of patients, acute phase reactants, rheumatoid factor, anticitrullinated peptide, previous conventional synthetic disease modifying antirheumatic drugs (csDMARD), initial and follow-up biological drugs. Disease activity of RA was measured by Disease Activity Score 28 (DAS-28) score. 

### 2.3. Rheumatoid Arthritis and Secondary Amyloidosis

Secondary amyloidosis was proven by renal biopsy. For renal involvement, serum creatinine and quantitative proteinuria was assessed before rituximab treatment. If available, serum creatinine, quantitative proteinuria, acute phase reactants, and DAS-28 score were recorded during follow-up period. Renal status was classified as improvement, stabilization, and aggravation. Improvement was defined as a sustained decrease in 24-h proteinuria and/or improved glomerular filtration rate (GFR) measured by the Cockcroft and Gault formula. Aggravation or treatment failure was defined as discontinuation of rituximab and/or increased GFR and/or proteinuria level. 

### 2.4. Collected Data of Rituximab Treatment

Rituximab treatment consisted of two endogenous infusions of 1 g per treatment cycle separated by a two-week interval (days 1 and 15), with repeated courses of therapy at least six months afterwards, depending on clinical response. All patients received premedication (100 mg methylprednisolone or equivalent) to prevent infusion reactions. Duration of follow-up after the first dose rituximab, course of rituximab, adverse events due to rituximab treatment, concomitant other drugs were also recorded. 

### 2.5. Statistical Analysis

Statistical analysis of the data was conducted using SPSS 18 (SPSS Inc., Chicago, IL, USA). Data are presented as mean ± standard deviation (SD) or range. We compared baseline characteristics and outcome measures after rituximab therapy.

This study was approved by Hacettepe University School of Medicine Ethical Committee for Clinical and Laboratory Research in May 2017 (KA-17/058).

## 3. Results

Of 1022 RA patients under biologic therapy, 0.7% (F/M: 5/2) patients had clinically apparent and histologically confirmed amyloidosis. We mainly assessed the efficacy and safety of rituximab and identified four patients received rituximab. The main clinical characteristics at the initiation of rituximab treatment and outcome data of these four patients are summarized in Table 1. 

Secondary AA amyloidosis is confirmed by renal biopsies in all of them. There was no diagnosis of Familial Mediterranean Fever (FMF). Laboratory values from the initial and last visit are in Table 1. All patients had received at least three DMARD before biologic therapy. In one patient rituximab (RTX) was the first line biologic agent, others were at least one TNF-i unresponsive. Rituximab was administered in combination with leflunomide in three cases and monotherapy in one patient.

Only one (P1) patient showed a significant decrease in proteinuria and disease activity. One patient (P3) showed a significant decrease in disease activity, stable renal function and proteinuria, but rituximab was switch to another agent because of recurrent bacterial infections. The other two patients (P2 and P4) were clinically unresponsive to rituximab, and patients were switched to another biologic agent. In one of these patients’ renal function was worsened, but this patient (P4) also had uncontrolled hypertension and diabetes mellitus, the other one (P2) showed a significant decrease in proteinuria and stable renal function (Table 1).

## 4. Discussion

In this study, the effects of rituximab on renal amyloidosis in RA patients could not be demonstrated due to the low number of patients analyzed and short follow-up period. The treatment of secondary AA amyloidosis in RA patients is controversial and still there is not an ideal therapy. In our study, decreased proteinuria in two patients (one was clinically unresponsive to rituximab) and positive results in literature data based on case reports suggest that rituximab may be an alternative option in some RA patients with amyloidosis [9,11,12].

Secondary AA amyloidosis is a devastating complication of chronic inflammatory diseases in which there is ongoing or recurring inflammation. Post-mortem incidence of amyloid complicating adult RA has generally ranged from 10 to 25 percent, similar values of 11 to 29 percent have been found with subcutaneous fat pad aspirates or gastrointestinal biopsies in living patients with RA, depending on population and diagnostic strategy [1,5]. However, the prevalence of clinically symptomatic amyloidosis was much lower [6]. In our biologic registry, we detected clinically symptomatic amyloidosis in 0.7% RA patients who were under biological therapy. 

The main goal for the treatment of AA amyloidosis is to control the activity of the underlying disorder. If untreated, secondary AA amyloidosis is a serious disease with a significant mortality due to end-stage renal disease, infection, heart failure, bowel perforation, or gastrointestinal bleeding [6]. TNF-α, IL-1, and IL-6 induce SAA production in hepatocytes, which is the precursor of AA amyloid fibrils [15]. These cytokines also take part in the pathogenesis of RA and furthermore, TNF-α also has other effects in the metabolism of SAA in macrophages contributing to tissue damage [16,17]. Although new drugs have proven to be significantly effective in the treatment of RA-related AA amyloidosis, no treatment modality has proven to be ideal. Recently, several isolated cases and small series have demonstrated therapeutic approaches focusing on TNF-i therapy or tocilizumab have achieved significant clinical improvement and partial resolution of AA amyloid deposits in RA patients [10,11,12]. Rituximab, an anti-CD-20 monoclonal antibody, is efficacious for patients with severe active RA who have exhibited an inadequate response to one or more TNF-i. However, to date, only in small case series preliminary clinical improvement has been shown with rituximab therapy for AA amyloidosis secondary to rheumatoid arthritis that is refractory to anti-TNF-α therapy [13,14].

A total of six RA patients with amyloidosis so far derived benefit from rituximab therapy in the literature. Also, five of these six patients were anti-TNF unresponsive as our cases (Only one patient used rituximab as a first line biologic agent because of advanced heart failure). All patients showed significant clinical improvement of the articular symptoms and marked reduction of the acute phase-reactants. Renal function or proteinuria remained stable or improved in 5 patients. (Table 2) [11,13,14].

Although the exact mechanism behind rituximab’s efficacy remains unknown, it may be an effective therapeutic option for serum AA amyloidosis refractory to TNF-i therapy.

The limitations of our study are mainly its retrospective design and low number of patients. Amyloidosis is a serious and rare complication of chronic inflammatory diseases and it is difficult to perform a controlled randomized trials and retrospective nature of the study was unavoidable. Although we tried to collect all cases of amyloidosis treated with rituximab and obtained all clinical data; retrospective design of our study might have resulted in missing some clinical/laboratory data and loss of follow-up of some patients with amyloidosis. Nevertheless, the results of this study reflect real life data of a large RA group. 

The effect of rituximab in RA patients with AA amyloidosis should be verified in well-designed future studies.

## Figures and Tables

**Table 1:** Characteristics of patients and efficacy of rituximab therapy by rheumatoid arthritis (RA) and secondary amyloid A (AA) amyloidosis.

	Patient 1	Patient 2	Patient 3	Patient 4
Sex/Age (years)	F/56	F/59	F/48	F/59
First symptom of disease (years)	22	44	6	31
Disease duration (years)	22	19	3	31
Clinical manifestations	RF-PU	PU, RF	PU	RF-PU
Previous therapy/treatment	MTX, SSZ, HCQ, LEF, CS, ETA, ADM	MTX, SSZ, LEF, CS	MTX, SSZ, LEF, CS, ADM, ETA	MTX, SSZ, LEF, CS, ETA
Comorbidity	-	HT	-	HT, DM
Romatoid factor (IU/mI) (0–20)	Positive	Positive	Negative	Negative
CCP (U/mI) (0–20)	N/A	N/A	N/A	Negative
Site of biopsy for amyloidosis	Renal	Renal	Renal	Renal
Duration after the first dose of RTX (months)	96	6	6	16
Concomitant therapy (DMARDs)	LEF, CS	CS	LEF, CS	LEF, CS
Other treatment for proteinuria	Losartan, Colchicine	Ramipril, Colchicine	Colchicine	Colchicine
Number of courses of therapy (1 g RTX separated by a 2-week interval)	16	1	1	3
Articular response and biochemical outcomes				
Baseline (Before the rituximab therapy)				
DAS 28-ESR	7.7	5.15	6.4	6.2
ESR (mm/h)	61	48	79	98
CRP (mg/dL)	6.5	0.54	3.81	1.69
Serum creatinine (mg/dL)	1.6	1.8	0.5	0.46
Quantitative proteinuria (g/L in 24 h)	4.8	4.5	2.4	0.5
Last follow-up				
DAS 28-ESR	4.49	6.28	3.47	5.2
ESR (mm/h)	13	61	15	87
CRP (mg/dL)	1.25	1.2	0.4	2.59
Serum creatinine (mg/dL)	1.56	1.93	0.7	6.8
Quantitative proteinuria/(g/L in 24 h)	0.4	0.3	2.3	15.5
Efficacy for the underlying disease	+	Active disease	+	Active disease
Efficacy for renal involvement	Improvement	Improvement	Stabilization	Renal failure, haemodialysis
Adverse events	NAE	NAE	Recurrent infections	NAE
Pursuit of RTX at the end of the follow-up	+	Switch to ETA	Switch to TCZ	Switch to TCZ

No adverse event; NAE, RF; rheumatoid factor, CCP; anti-cyclic citrullinated peptide, DAS-28; Disease Activity Score 28, ESR; sedimentation, CRP; C-reactive protein, RTX; rituximab, DMARDs; disease-modifying antirheumatic drugs, renal failure; RF, proteinuria; PU, HT; hypertension, DM; diabetes mellitus, SSZ; sulfasalazine, HCQ; hydroxychloroquine, LEF; leflunomide, CS; corticosteroids, ETA; etanercept, ADM; adalimumab, TCZ; tocilizumab, F; female.

**Table 2:** Efficacy of rituximab therapy in RA and AA amyloidosis: Briefly; A review of the literature.

Author (Reference)/Year of Publication	Age/Sex	Disease Duration (Years)	Treatment before RTX	Concomitant Therapy	Follow-Up (Months)	Creatinine (Initial-Last) (mg/dL)	Proteinuria (Initial-Last) (g/day)	CRP (Initial-Last) (mg/L)	DAS 28 (Initial-Last)	Efficacy for Underlying Diseases
Narváez et al. 2010 [14]	F/46	14	AZA, CQ, CsA, LEF, MTX, IFX, ETA, ADM	-	41 (7 cycle)	1.05–1.09	0.44–0.15	110–18	7.68–5.24	+
	F/75	28	GS, CQ, DP, LEF, MTX, SSZ, IFX,	-	22 (2 cycle)	2.52–2.58	0.94–1.22	12.6–9.5	6.53–3.35	+
	F/56	40	GS, DP, CQ, LEF, MTX, ADM	MTX 12.5 mg/w	15 (3 cycle)	2.16–2.10	0.29–0.19	15.8–6.5	5.72–3.98	+
	F/67	16	GS, CQ, SSZ, MTX, IFX,	MTX 10 mg/w	12 (3 cycle)	1.99–1.58	1.75–0.65	17–3.6	7.81–4.35	+
Burkart et al. 2012 [13]	F/61	34	(DMARD therapy N/A) ETA, ADM	N/A	18	N/A	N/A	N/A	N/A	+
Pamuk et al. 2013 [11]	M/50	11	CS, SSZ, HCQ	N/A	10	2.7–1.8	1.4–0.3	N/A	N/A	+
Our study Kilic et al.	F/56 (P1)	22	MTX, SSZ, HCQ, LEF, CS, ETA, ADM	LEF 20 mg/d CS 5 mg/d	96	1.6–1.56	4.8–0.4	65–12	7.7–4.49	+
	F/59 (P2)	19	MTX, SSZ, LEF, CS	CS 5 mg/d	6	1.8–1.9	4.5–0.3	5.4–12	5.1–6.2	-

ADM: adalimumab; AZA: azathioprine; CQ: chloroquine; CRP: C-reactive protein; CS: corticosteroid; CsA: ciclosporin A; DAS28: disease activity score in 28 joints; DP: D-penicillamine; ESR: erythrocyte sedimentation rate; ETA: etanercept; GS: gold salts; HCQ: hydroxychloroquine; IFX: infliximab; MTX: methotrexate; LEF; leflunomide; SSZ: sulfasalazine. DMARD: disease modifying anti-rheumatic drugs; N/A: Not available.
